# Highly efficient eco-friendly X-ray scintillators based on an organic manganese halide

**DOI:** 10.1038/s41467-020-18119-y

**Published:** 2020-08-28

**Authors:** Liang-Jin Xu, Xinsong Lin, Qingquan He, Michael Worku, Biwu Ma

**Affiliations:** 1 Department of Chemistry and Biochemistry, Florida State University, Tallahassee, FL 32306 USA; 2 Materials Science and Engineering Program, Florida State University, Tallahassee, FL 32306 USA

**Keywords:** Materials chemistry, Materials for devices, Materials for optics

## Abstract

Scintillation based X-ray detection has received great attention for its application in a wide range of areas from security to healthcare. Here, we report highly efficient X-ray scintillators with state-of-the-art performance based on an organic metal halide, ethylenebis-triphenylphosphonium manganese (II) bromide ((C_38_H_34_P_2_)MnBr_4_), which can be prepared using a facile solution growth method at room temperature to form inch sized single crystals. This zero-dimensional organic metal halide hybrid exhibits green emission peaked at 517 nm with a photoluminescence quantum efficiency of ~ 95%. Its X-ray scintillation properties are characterized with an excellent linear response to X-ray dose rate, a high light yield of ~ 80,000 photon MeV^−1^, and a low detection limit of 72.8 nGy s^−1^. X-ray imaging tests show that scintillators based on (C_38_H_34_P_2_)MnBr_4_ powders provide an excellent visualization tool for X-ray radiography, and high resolution flexible scintillators can be fabricated by blending (C_38_H_34_P_2_)MnBr_4_ powders with polydimethylsiloxane.

## Abstract (web summary)

Scintillation-based X-ray detection is promising for applications in various areas ranging from security to healthcare, and low-cost and eco-friendly scintillation materials would be beneficial. Here the authors report a facile solution growth of organic manganese halide for efficient X-ray scintillation.

## Introduction

Scintillators, with the ability to convert ionizing radiation into visible photons, have received extensive attention in recent years, as they can be used as radiation detectors for radiation exposure monitoring, security inspection, space exploration, and medical imaging^1–4^. While various types of materials have been used for X-ray scintillators, there are still many issues and limitations to existing organic and inorganic scintillation materials, for example, rigorous conditions required for the preparation of inorganic crystals and their hygroscopicity, anisotropic scintillation of organic crystals, low light yields in plastics, and so on^5–9^. Therefore, searching for low-cost, high-performance scintillation materials is still of great scientific and practical interest.

Recently, lead halide perovskites, such as CsPbBr_3_ and MAPbBr_3_, have been demonstrated in direct X-ray imaging, owing to their strong X-ray absorption and efficient conversion to charge carriers^10–20^. X-ray scintillators have also been developed using highly emissive metal halide perovskite nanocrystals^21–26^. However, the toxicity of lead in these halide perovskites might limit their potential commercial applications. In this regard, lead-free metal halide perovskites and hybrids with efficient charge extraction and high photoluminescence quantum efficiencies (PLQEs) for X-ray detectors have received increasing interests^27–33^. For instance, Tang’s group reported sensitive X-ray detectors using double perovskite Cs_2_AgBiBr_6_ single crystals^31^. More recently, the use of low-dimensional metal hybrids, such as Bmpip_2_SnBr_4_ and Rb_2_CuX_3_ (X = Cl and Br), for scintillators has been demonstrated by Kovalenko’s and Tang’s groups, respectively^32–34^.

Eco-friendly organic manganese (II) halide hybrids have been reported to exhibit strong photoluminescence (PL) with colors ranging from green to red^35–40^. The luminescence mechanisms of this class of materials are well known, that is, from the d–d transitions in tetrahedral and octahedral crystal fields for green and red emissions, respectively^37,41^. For their excellent optical properties, various applications have been demonstrated using organic manganese (II) halide hybrids. For instance, organic light-emitting diodes with external quantum efficiencies of ~10% have been reported by using tetrabromide manganese (II) complex (PPh_4_)_2_MnBr_4_^35^ and dibenzofuran-based phosphine oxide manganese (II) bromides (DBFDPO-MnBr_2_)^42^. Luminescent vapochromism has also been realized via the reversible conversion of ligand fields in diphenylphosphine oxide-based manganese (II) hybrids MnBr_2_(dppeO_2_)^38^. A selective fluorescent sensor for different organic solvents was recently demonstrated using (C_9_NH_20_)_2_MnBr_4_^36^. To the best of our knowledge, the attempt on scintillators based on organic manganese (II) halide hybrids has not been reported yet.

Here, we demonstrate high-performance eco-friendly X-ray scintillators based on a 0D phosphonium manganese (II) bromide hybrid (C_38_H_34_P_2_)MnBr_4_. This organic manganese (II) halide hybrid can be easily prepared by using low-cost commercially available raw materials via a facile room-temperature solvent diffusion method with excellent repeatability and large scalability. High-quality (C_38_H_34_P_2_)MnBr_4_ single crystals with sizes of >1 in. show great thermal stability and bright green emission peaked at 517 nm with a PLQE of ~95%. Scintillators based on (C_38_H_34_P_2_)MnBr_4_ display great performance with exceptional linearity, high light yield, and low detection limits, which enable high-resolution X-ray images.

## Results

### Synthesis and characterization

**Fig. 1:**
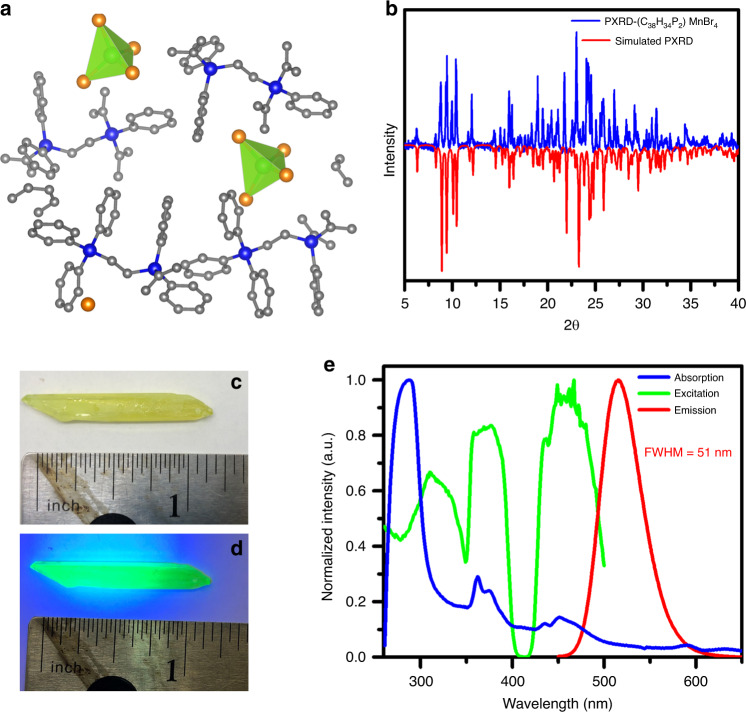
Structural and photophysical characterization of (C_38_H_34_P_2_)MnBr_4_ single crystals. **a** View of the single-crystal structure of (C_38_H_34_P_2_)MnBr_4_ (Mn green, Br orange, P blue, C gray; hydrogen atoms were hidden for clarity). **b** PXRD patterns of (C_38_H_34_P_2_)MnBr_4_ and the corresponding simulated peaks from the single-crystal structure. The images of a (C_38_H_34_P_2_)MnBr_4_ single crystal under daylight (**c**) and UV light (**d**). **e** Absorption, excitation, and emission spectra of (C_38_H_34_P_2_)MnBr_4_.

Similar to other low-dimensional organic metal halide hybrids reported by our group^28–30^, 0D (C_38_H_34_P_2_)MnBr_4_ single crystals were obtained by diffusing diethyl ether into a dichloromethane (DCM) precursor solution containing ethylenebis(triphenylphosphonium bromide) (C_38_H_34_P_2_Br_2_) and MnBr_2_ in a ratio of 1:1. The details of synthesis and purification could be found in Supplementary Scheme 1 and Fig. 1. The crystal structure of (C_38_H_34_P_2_)MnBr_4_ single crystals was determined by single-crystal X-ray diffraction (SCXRD). As shown in Fig. 1a and Supplementary Fig. 2, (C_38_H_34_P_2_)MnBr_4_ crystallizes at a monoclinic space group of *C*_2/*c*_, possessing 0D structure at the molecular level with MnBr_4_ tetrahedrons isolated and surrounded by C_38_H_34_P_2_^2+^ cations. The manganese center adopts a typical tetra-coordinated geometry bonded to bromide ions, with an average Mn–Br bond length of 2.51 Å and bond angle of 108.48° (Supplementary Tables 1 and 2), similar to those of previously reported MnBr_4_ complexes^35^. The powder XRD pattern of (C_38_H_34_P_2_)MnBr_4_ powder is identical to the simulated result from SCXRD data (Fig. 1b), suggesting the high phase purity of the as-prepared single crystals. No weight loss was observed before 310 °C in thermogravimetric analysis (TGA) as shown in Supplementary Fig. 3, suggesting a high thermal stability. The differential scanning calorimetry (DSC) results with an endothermic peak at 295 °C (Supplementary Fig. 3), which could be the melting point of (C_38_H_34_P_2_)MnBr_4_, suggest its high phase stability at elevated temperatures below 295 °C.

### Photophysical properties

The (C_38_H_34_P_2_)MnBr_4_ single crystals are pale green under ambient light and become highly emissive upon irradiating with ultraviolet (UV) light as shown in Fig. 1c, d. The photophysical properties were further investigated using UV–vis absorption and steady-state PL spectroscopies. As shown in Fig. 1e, (C_38_H_34_P_2_)MnBr_4_ exhibits an intense absorption band around 285 nm along with two absorption peaks at 360 and 450 nm. The excitation spectrum has the same features as the absorption spectrum in a low-energy band, which are corresponding to two groups of transitions: ^6^A_1_ → ^4^G and ^6^A_1_ → ^4^D. (See the optical transitions in tetrahedrally coordinated Mn^2+^ ion in Supplementary Scheme 2.) Upon irradiation in the range of 300–400 nm, bright green emission peaked at 517 nm was observed with a full-width at half-maximum of 51 nm, a high PLQE of ~95%, and a long single-exponential decay lifetime of 318 µs (*R*^2^ = 0.999) (Supplementary Fig. 4). The strong green emission is well known to be from d–d ^4^T_1_ → ^6^A_1_ transition of Mn^2+^ ion with a tetrahedral coordination geometry. Moreover, (C_38_H_34_P_2_)MnBr_4_ demonstrated great moisture stability with PL intensity unchanged after exposure in an ambient atmosphere for 1 month (Supplementary Fig. 5). The high emission efficiency together with good quality of facilely prepared single crystals suggest the suitability of (C_38_H_34_P_2_)MnBr_4_ for luminescent devices.

### X-ray scintillation properties

**Fig. 2:**
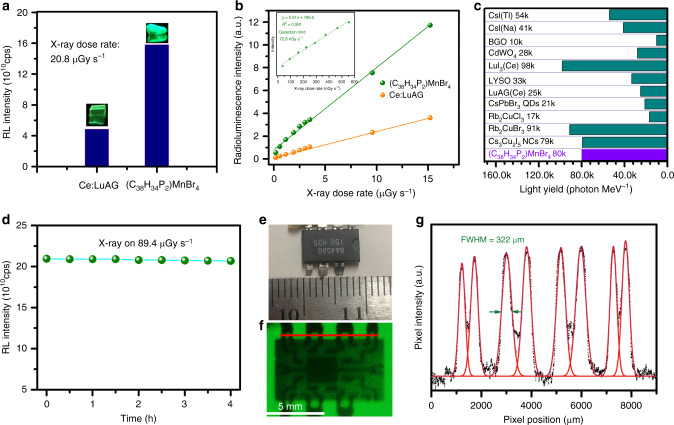
X-ray scintillation properties of (C_38_H_34_P_2_)MnBr_4_. **a** Comparison of RL intensities for the standard reference Ce:LuAG and (C_38_H_34_P_2_)MnBr_4_ under dose rate of 20.8 μGy s^−1^. The inset shows the corresponding images under the same X-ray irradiation. **b** Dose rate dependence of the RL intensity of standard reference Ce:LuAG and (C_38_H_34_P_2_)MnBr_4_. The inset shows the detection limit measurement under low X-ray dose for (C_38_H_34_P_2_)MnBr_4_. The detection limit can be achieved when the RL intensity is three times higher than the background intensity. **c** Comparison of scintillator light yields of (C_38_H_34_P_2_)MnBr_4_ and previously reported and commercially available scintillators. **d** The change of the RL intensity under continuous X-ray excitation with a dose rate of 89.4 μGy s^−1^. **e** Image of a speaker chip under bright-field. **f** The X-ray images of the speaker chip by using (C_38_H_34_P_2_)MnBr_4_ scintillator screen, acquired with a digital camera. **g** Spatial resolution measurement by the fitting of intensity spread profile with Gaussian function. The FWHM was taken as resolution. The red line in **f** shows the data trace of collection.

To explore the scintillation performance of (C_38_H_34_P_2_)MnBr_4_, a commercially available scintillation material, cerium-doped lutetium aluminum garnet (Ce:LuAG), was used as a standard reference as it exhibits a similar PL emission peaked at ~520 nm that could minimize the influence of response difference by detectors. The X-ray radioluminescence (RL) spectra of (C_38_H_34_P_2_)MnBr_4_ and Ce:LuAG were obtained by using Edinburgh FS5 fluorescence spectrophotometer equipped with a X-ray generator (Amptek Mini-X tube, Au target, 4 W). As shown in Supplementary Fig. 6, both RL emissions are identical to their PL emissions. Interestingly, the RL intensity of (C_38_H_34_P_2_)MnBr_4_ is >3 times higher than that of Ce:LuAG under the same X-ray dose rate irradiation. Moreover, the X-ray image of (C_38_H_34_P_2_)MnBr_4_ single crystals is much brighter than that of Ce:LuAG, as shown in Fig. 2a, suggesting that (C_38_H_34_P_2_)MnBr_4_ is more sensitive to X-ray irradiation than Ce:LuAG. To evaluate the scintillator response to X-ray dose rate, the RL intensities were measured under various X-ray dose rates for (C_38_H_34_P_2_)MnBr_4_ and Ce:LuAG. Figure 2b and Supplementary Fig. 7 show that both scintillators exhibit excellent linearities to the X-ray dose rates in a large range from 36.7 nGy s^−1^ to 89.4 μGy s^−1^. Moreover, (C_38_H_34_P_2_)MnBr_4_ exhibits a higher response to X-ray dose than Ce:LuAG with a larger slope. The reproducibility of the responses to X-ray for (C_38_H_34_P_2_)MnBr_4_ was validated by using single crystals with different sizes and shapes. Almost the same sensitivity was recorded for all the samples, as shown in Supplementary Fig. 8. The detection limit of X-ray dose rate was derived to be 72.8 nGy s^−1^ for (C_38_H_34_P_2_)MnBr_4_ when the signal-to-noise ratio (SNR) is 3, which is ~75 times lower than the dose rate required for X-ray diagnostics (5.5 μGy s^−1^)^12^. Light yield is another important parameter to evaluate the performance of scintillators, which is dependent on the amplitude of X-ray response and the RL spectra. Since the X-ray dose response of (C_38_H_34_P_2_)MnBr_4_ is 3.2 times higher than that of Ce:LuAG (with a light yield of 25,000 photon MeV^−1^) and they have a similar RL spectrum, the light yield of (C_38_H_34_P_2_)MnBr_4_ could be derived to be ~79,800 photon MeV^−1^. As shown in Fig. 2c, the light yield of (C_38_H_34_P_2_)MnBr_4_ is comparable to those of recently reported lead-free metal halides, such as Cs_3_Cu_2_I_5_ (79,279 photon MeV^−1^)^43^ and Rb_2_CuBr_3_ (91,056 photon MeV^−1^)^33^, and much better than those of Rb_2_CuCl_3_ (16,600 photon MeV^−1^)^34^, widely investigated CsPbBr_3_ nanocrystals (21,000 photon MeV^−1^)^22^, and many commercially available scintillators, such as CsI:Tl (54,000 photon MeV^−1^) and CdWO_4_ (28,000 photon MeV^−1^). Moreover, based on the toxicity classification (health and environment) information of metal halides from material safety data sheet, (C_38_H_34_P_2_)MnBr_4_ is believed to be significantly less toxic than existing scintillators mentioned above. As shown in Supplementary Table 3, Pb(II), Cu(I), CsI, and GdWO_4_ possess the most severe toxicity in the environment, and Tl(I) and CsI are moderately toxic to health. Also, ^87^Rb isotope is radioactive^34^. Mn(II) is considered to be less toxic for health and friendly to the environment. The stability of (C_38_H_34_P_2_)MnBr_4_ single crystals against X-ray irradiation was evaluated by monitoring the changes of RL intensity under continuous X-ray irradiation with a dose rate of 89.4 μGy s^−1^. Figure 2d shows that little-to-no radio-degradation was observed after 4 h exposure to X-ray irradiation, suggesting high stability for scintillator applications.

### X-ray imaging

To further validate the potential of (C_38_H_34_P_2_)MnBr_4_ as scintillation material for practical X-ray imaging, a home-built X-ray imaging system was constructed, as shown in Supplementary Fig. 9. The scintillator screen was prepared by refilling the glass holder with (C_38_H_34_P_2_)MnBr_4_ fine powders with the particle size <3 µm (see scanning electron microscope (SEM) images in Supplementary Fig. 10). A speaker chip with a size of 9 mm × 6 mm, as shown in Fig. 2e, was used as a target placed between the X-ray source and the scintillator screen for X-ray image. The configuration inside of the chip cannot be seen directly with our eyes, which however could be revealed clearly by X-ray imaging using a (C_38_H_34_P_2_)MnBr_4_-based scintillator (Fig. 2f). The large difference in X-ray absorption for different materials in the chip resulted in spatial intensity contrast displayed in the scintillator screen. The spatial resolution was calculated as 0.322 mm by fitting the point spread function of the intensity profile (Fig. 2g). Image contrast is another important parameter for practical imaging applications; image lag or ghosting would happen if the emission with a long lifetime has a strong afterglow after X-ray being turned off. To exclude the effect of afterglow, we measured the afterglow intensities of (C_38_H_34_P_2_)MnBr_4_, as shown in Supplementary Fig. 11. The intensity decreased to the background level in 10 ms after the cease of the excitation source, indicating the suitability for high contrast imaging. The excellent performance of X-ray imaging could be attributed to the negligible self-absorption, high PLQE, light yield, and low detection limit of (C_38_H_34_P_2_)MnBr_4_^33,34,43,44^.

**Fig. 3:**
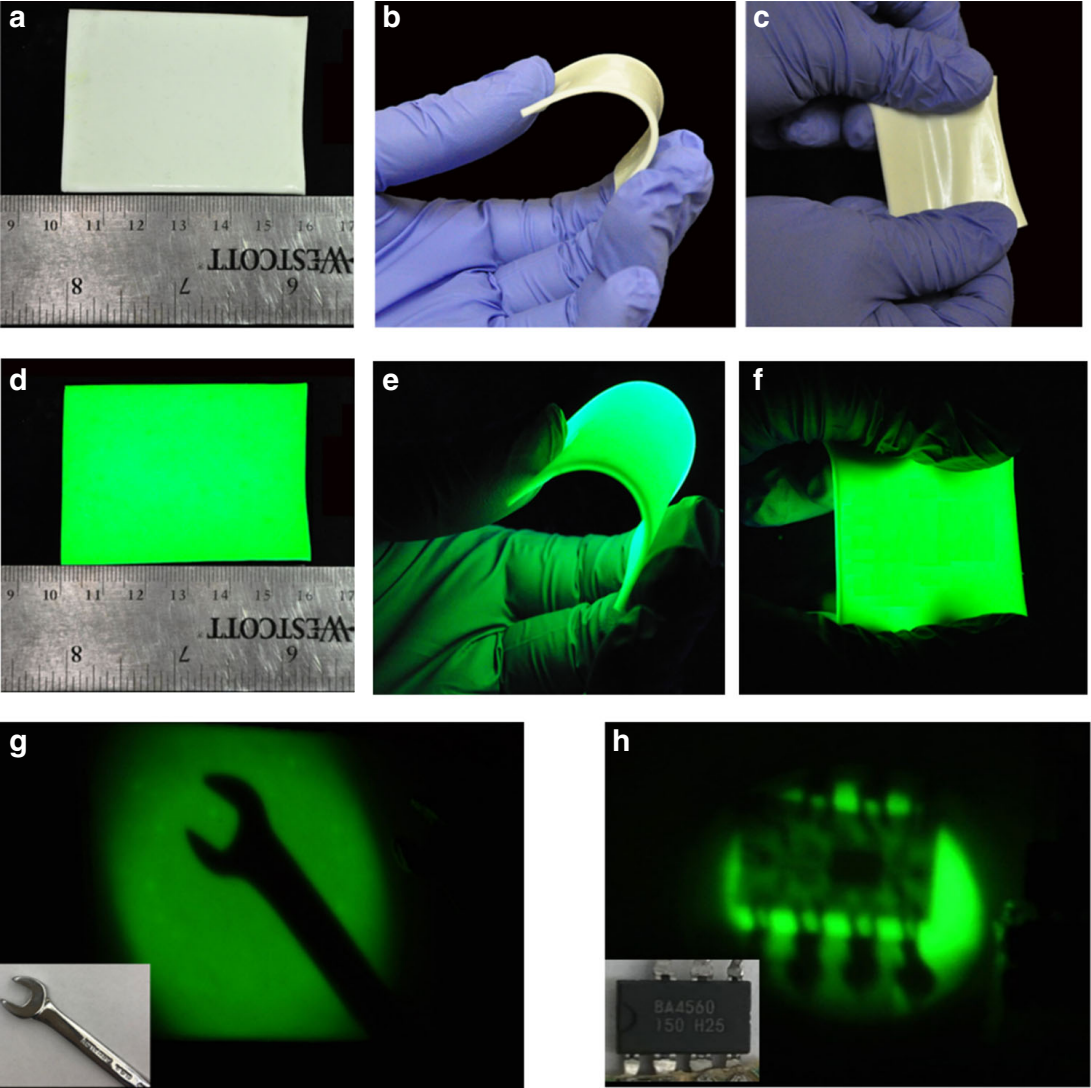
Flexible X-ray scintillator screens. The photographs of a flexible scintillator screen based on (C_38_H_34_P_2_)MnBr_4_ **a** under flatting, **b** under bending stress, **c** under stretching, under ambient light. The photographs of a flexible scintillator screen based on (C_38_H_34_P_2_)MnBr_4_ under UV excitation **d** under flatting, **e** under bending stress, **f** under stretching. **g** X-ray image of a wrench by using a flexible (C_38_H_34_P_2_)MnBr_4_ scintillator screen, inset shows the wrench used for scanning. **h** X-ray image of a speaker chip by using a flexible (C_38_H_34_P_2_)MnBr_4_ scintillator screen, inset shows the speaker chip used for scanning.

Flexible devices have received tremendous attention nowadays for their good foldability, high crack resistance, favorable compatibility, and potential application in portable and wearable devices. Here, flexible scintillators with large size (4.5 × 5.8 cm^2^) were demonstrated by blending (C_38_H_34_P_2_)MnBr_4_ fine powders with polydimethylsiloxane (PDMS). As shown in Fig. 3a–c, the resulting films show excellent flexibility, which can be easily bent and stretched. Moreover, the film shows high uniformity and strong emission under UV irradiation (Fig. 3d–f). The scintillation performance of flexible scintillation screens was characterized as shown in Supplementary Fig. 12, which exhibit excellent linearities to the X-ray dose rates in a large range from 36.7 nGy s^−1^ to 89.4 μGy s^−1^, with a slightly lower light yield (66,256 photon MeV^−1^) and detection limit (461.1 nGy s^−1^), as compared to those of single crystals. This is not surprising, considering that the content of (C_38_H_34_P_2_)MnBr_4_ is reduced in the blends, the distribution of (C_38_H_34_P_2_)MnBr_4_ might not be perfectly uniform in the blends, and PDMS could also affect the X-ray absorption. To demonstrate the capability of the X-ray imaging, a wrench and a speaker chip were scanned as the targets (Fig. 3g, h). Distinct color contrast and detail inside of the chip can be displayed in the flexible film with good resolution.

## Discussion

In conclusion, a new 0D organic manganese (II) halide hybrid (C_38_H_34_P_2_)MnBr_4_ has been developed to exhibit highly efficient green emission upon photo and X-ray excitations. Single crystals with sizes of >1 in. could be prepared via a facile solution growth method at room temperature, which shows remarkable scintillation properties with excellent response linearity to dose rate, high light yield, and low detection limits. The X-ray scintillation characteristics were found to be superior to those of metal halide perovskite nanocrystals and most of today’s commercially available scintillators. X-ray imaging was also successfully demonstrated with high resolution. The low-cost, facile preparation, environmentally friendly, and state-of-the-art scintillation performance make this organic manganese (II) hybrid (C_38_H_34_P_2_)MnBr_4_ a highly promising scintillator for commercial applications. Our work paves a new way to explore new low-cost, high-performance eco-friendly hybrid materials for radiation scintillators.

## Methods

### Materials

Manganese (II) bromide, and ethylenebis(triphenylphosphonium bromide) were purchased from Sigma-Aldrich. Dimethylformamide (99.8%), DCM (99.9%), and diethyl ether (Et_2_O, 99.8%) was purchased from VWR. Standard scintillator Ce:LuAG was purchased from Jiaxing AOSITE Photonics Technology Co., Ltd. All reagents and solvents were used without further purification unless otherwise stated.

### Growth of 0D (C_38_H_34_P_2_)MnBr_4_ single crystals

MnBr_2_ (429.5 mg, 2.0 mmol) and ethylenebis(triphenylphosphonium bromide) (1.424 g, 2.0 mmol) were dissolved in 10 mL DCM solution and then filtered into a 20 mL vial to form a clear precursor solution. Then, the vial was placed in a 100 mL vial with 60 mL Et_2_O inside. The as-prepared solution was sealed and left to stand for ~3 days to afford pale green block crystals. Yield ~89%.

### Scintillator screen

First, (C_38_H_34_P_2_)MnBr_4_ single crystals were hand-ground to fine powders by using mortar and pestle. Then, the scintillator screen was prepared by filling the fine powder into the PXRD holder. The flexible screen was prepared by blending the powder with a two-part PDMS EI-1184 at a mass ratio of 40%. The mixture gel was placed in a polytetrafluoroethylene mold and cured at 100 °C for 30 min in a muffle furnace and then the flat and smooth film was formed after cooling down the mixture gel to room temperature.

### Single-crystal X-ray diffraction

Single-crystal X-ray data for the (C_38_H_34_P_2_)MnBr_4_ hybrid were collected using a Rigaku XtaLAB Synergy-S diffractometer equipped with a HyPix-6000HE Hybrid Photon Counting (HPC) detector and dual Mo and Cu microfocus sealed X-ray source.

### Powder X-ray diffraction

The PXRD analysis was performed on Panalytical X’PERT Pro Powder X-Ray Diffractometer using Copper X-ray tube (standard) radiation at a voltage of 40 kV and 40 mA, and X’Celerator RTMS detector. The diffraction pattern S was scanned over the angular range of 5–40° (2*θ*) with a step size of 0.02, at room temperature.

### Absorption spectrum measurements

Absorption spectra of (C_38_H_34_P_2_)MnBr_4_ hybrid were measured at room temperature on Cary 5000 UV–Vis-NIR spectrophotometer.

### PL steady-state studies

Steady-state PL spectrum of (C_38_H_34_P_2_)MnBr_4_ was obtained at room temperature on an FS5 spectrofluorometer (Edinburgh Instruments).

### Photoluminescence quantum efficiency

The PLQEs were acquired using a Hamamatsu Quantaurus-QY Spectrometer (Model C11347-11) equipped with a xenon lamp, integrated sphere sample chamber, and CCD detector. The PLQEs were calculated by the equation: η_QE_ = I_S_/(E_R_ − E_S_), in which I_S_ represents the luminescence emission spectrum of the sample, E_R_ is the spectrum of the excitation light from the empty integrated sphere (without the sample), and E_S_ is the excitation spectrum for exciting the sample.

### Time-resolved PL

Time-resolved emission data were collected at room temperature using the Edinburgh FLS920 fluorescence spectrometer. The dynamics of emission decay were monitored by using the time-correlated single-photon counting capability with data collection for 10,000 counts. The average lifetime was obtained by the single-exponential fitting.

### Afterglow intensity measurement

The afterglow intensity was recorded by continuously irradiating 20 s under xenon lamp and then the afterglow signal was collected by Hamamatsu R928 PMT with the time interval of 10 ms.

### Thermogravimetric analysis and differential scanning calorimetry

TGA and DSC were carried out using a TA instruments Q600 system. The samples were heated from room temperature to 700 °C at a rate of 5 °C min^−1^, under a nitrogen flux of 100 mL min^−1^.

### RL and X-ray imaging

The RL spectra were acquired by using an Edinburgh FS5 spectrofluorometer (Edinburgh Instruments) equipped with an X-ray source (Amptek Mini-X tube with an Au target and 4 W maximum power output). The X-ray response intensity was examined and collected by a Hamamatsu R928 PMT. The scintillator light yield was estimated using the following equation. Here, the Ce:LuAG was used as the reference with a known light yield of 25,000 photon MeV^−1^. The spectrum of (C_38_H_34_P_2_)MnBr_4_ is similar to that of Ce:LuAG after correcting the intensity and wavelength from the correction files of R928 PMT. Then, the light yield could be estimated by comparing the corrected response amplitude (*R*) of the two samples using Eq. (1):

1 $$\frac{{{\mathrm{LY}}_{{\mathrm{C}}_{38}{\mathrm{H}}_{34}{\mathrm{P}}_2{\mathrm{MnBr}}_4}}}{{{\mathrm{LY}}_{{\mathrm{Ce}}:{\mathrm{LuAG}}}}} = \frac{{{\mathrm{R}}_{{\mathrm{C}}_{38}{\mathrm{H}}_{34}{\mathrm{P}}_2{\mathrm{MnBr}}_4}}}{{{\mathrm{R}}_{{\mathrm{Ce}}:{\mathrm{LuAG}}}}} \times \frac{{\smallint {\mathrm{I}}_{{\mathrm{Ce}}:{\mathrm{LuAG}}}\left( {\uplambda} \right){\mathrm{S}}\left( {\uplambda} \right){\mathrm{d}}\lambda /\smallint {\mathrm{I}}_{{\mathrm{Ce}}:{\mathrm{LuAG}}}\left( {\uplambda} \right){\mathrm{d}}\lambda }}{{\smallint {\mathrm{I}}_{{\mathrm{C}}_{38}{\mathrm{H}}_{34}{\mathrm{P}}_2{\mathrm{MnBr}}_4}\left( {\uplambda} \right){\mathrm{S}}\left( {\uplambda} \right){\mathrm{d}}\lambda /\smallint {\mathrm{I}}_{{\mathrm{C}}_{38}{\mathrm{H}}_{34}{\mathrm{P}}_2{\mathrm{MnBr}}_4}\left( {\uplambda} \right){\mathrm{d}}\lambda }}.$$

The radiation dose rate of the X-ray source was calibrated by using an ion chamber dosimeter. The X-ray images were acquired by using a digital camera (Nikon D90).

### Scanning electron microscopy

The particle size of (C_38_H_34_P_2_)MnBr_4_ fine powders were investigated by FEI Nova NanoSEM 400 SEM.

## Supplementary information

Supplementary Information

Peer Review File

## Data Availability

The data that support the findings of this study are available from the corresponding author on reasonable request. The X-ray crystallographic data for this paper has been deposited at the Cambridge Crystallographic Data Centre (CCDC), under deposition number 1972108. These data can be obtained free of charge from the CCDC via www.ccdc.cam.ac.uk/data_request/cif.
