# Supplementary material for: Highly efficient eco-friendly X-ray scintillators based on an organic manganese halide
Source: Nat Commun. 2020 Aug 28;11:4329. doi: 10.1038/s41467-020-18119-y (PMC7455565; doi:10.1038/s41467-020-18119-y)
Supplement: Supplementary file 1 — Supplementary Information [file 41467_2020_18119_MOESM1_ESM.pdf]

Supplementary Table 1 | Single crystal X-ray diffraction data of $(C_{38}H_{34}P_2)MnBr_4$.

Compound	$(C_{38}H_{34}P_2)MnBr_4$
Empirical formula	$C_{38}H_{34}Br_4P_2Mn$
Molecular weight	927.13
Temperature/K	293(2)
Crystal system	monoclinic
Space group	$C2/c$
a/Å	10.108(3)
b/Å	18.594(5)
c/Å	20.176(6)
α/°	90
β/°	99.860(4)
γ/°	90
Volume/Å^3	3736.0(19)
Z	4
ρ_{calc}g/cm^3	1.648
μ/mm^{-1}	4.743
R_1, wR_2	0.0305[a], 0.1110[b]
Goodness-of-fit on F^2	0.835

a) $R1 = \Sigma \| F_o | - | F_c \| / \Sigma | F_o \|$. b) $wR2 = [\Sigma w(F_o^2 - F_c^2)^2 / \Sigma w(F_o^2)^2]^{1/2}$

Supplementary Table 2 | Selected bond length and bond angle of $(C_{38}H_{34}P_2)MnBr_4$.

Bond	Distance (Å)
Mn1-Br1	2.505
Mn1-Br2	2.520

Bonds	Angle (°)
Br1-Mn1-Br1A	101.6
Br1-Mn1-Br2	113.5
Br1-Mn1-Br2A	106.8
Br2-Mn1-Br2A	114.0

Supplementary Table 3 | Material toxicity data.

Materials	Acute toxicity (Health)	Acute aquatic hazard (Environment)
$PbBr_2$	Category 4	Category 1
CuCl	Category 4	Category 1
TlBr	Category 2	Category 2
CsI	Category 2	Category 1
$CdWO_4$	Category 4	Category 1
$MnBr_2$	Category 4	Contains no substances known to be hazardous to the environment
TPPBr (Tetraphenylphosphonium bromide)	Not a hazardous substance	Not a hazardous substance

The toxicity classification (health and environment) of the metal halides acquired from material safety data sheet (MSDS). Acute toxicity category 1 represents the most severe toxicity with $LD_{50} < 5$ mg/kg in oral, and category 2 with $5 < LD_{50} < 50$ mg/kg, category 3 with $50 < LD_{50} < 300$ mg/kg and category 4 with $300 < LD_{50} < 2000$ mg/kg. As the toxicity classification of the reported and commercially available scintillator are unknow, we compare the toxicity of metal halides used for scintillators synthesis for convenience. Also, we refer to the toxicity of tetraphenylphosphonium bromide instead of ethylenebis-triphenylphosphonium bromide, as its MSDS data is unavailable.

Supplementary Scheme 1 | The synthesis of the 0D ($C_{38}H_{34}P_2$)$MnBr_4$.

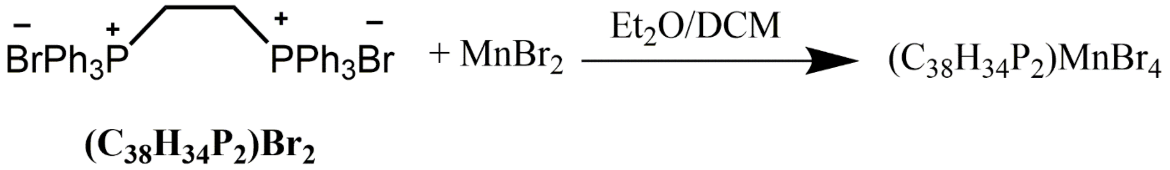

Supplementary Scheme 2 | Schematic diagram showing the energy adsorption, migration, and emission process in tetrahedrally coordinated Mn^{2+} ion.

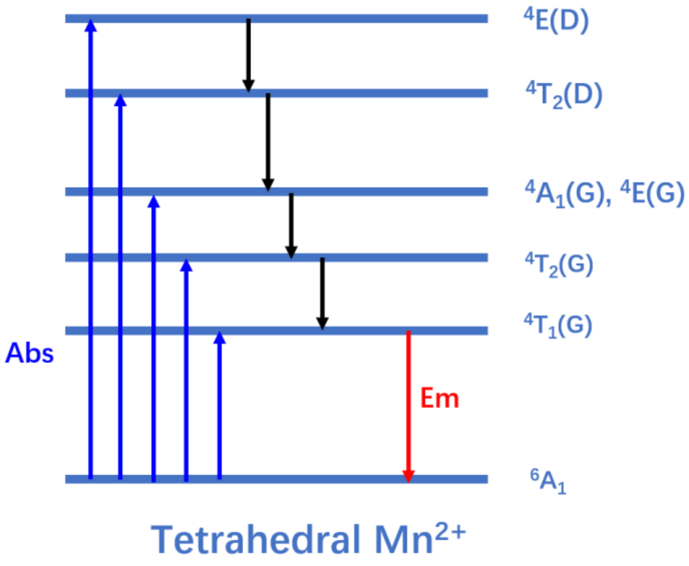

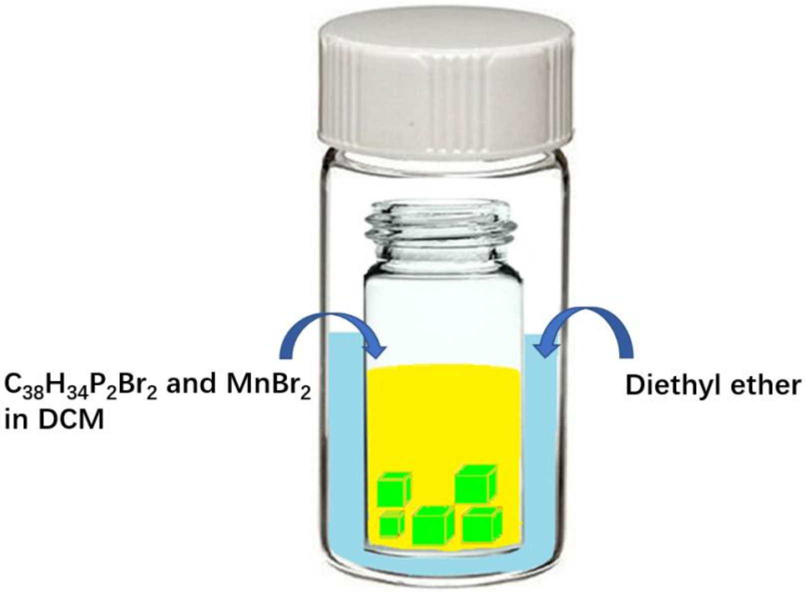

Supplementary Figure 1 | Illustration of solution crystal growth of $(C_{38}H_{34}P_2)MnBr_4$.

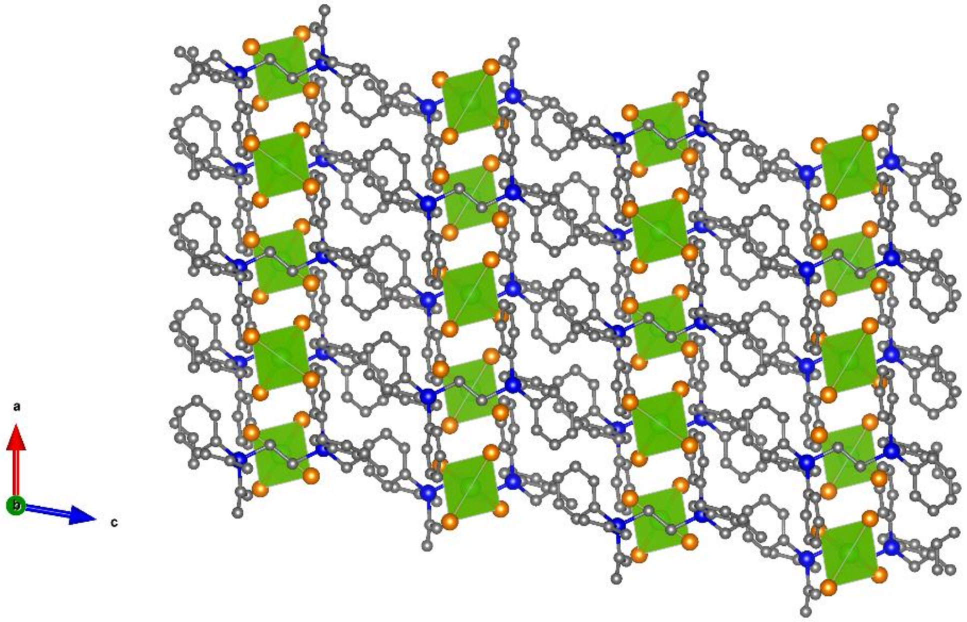

Supplementary Figure 2 | Crystal structure of ($C_{38}H_{34}P_2$)$MnBr_4$ (Mn green, Br orange, P blue, C gray; hydrogen atoms were hidden for clarity).

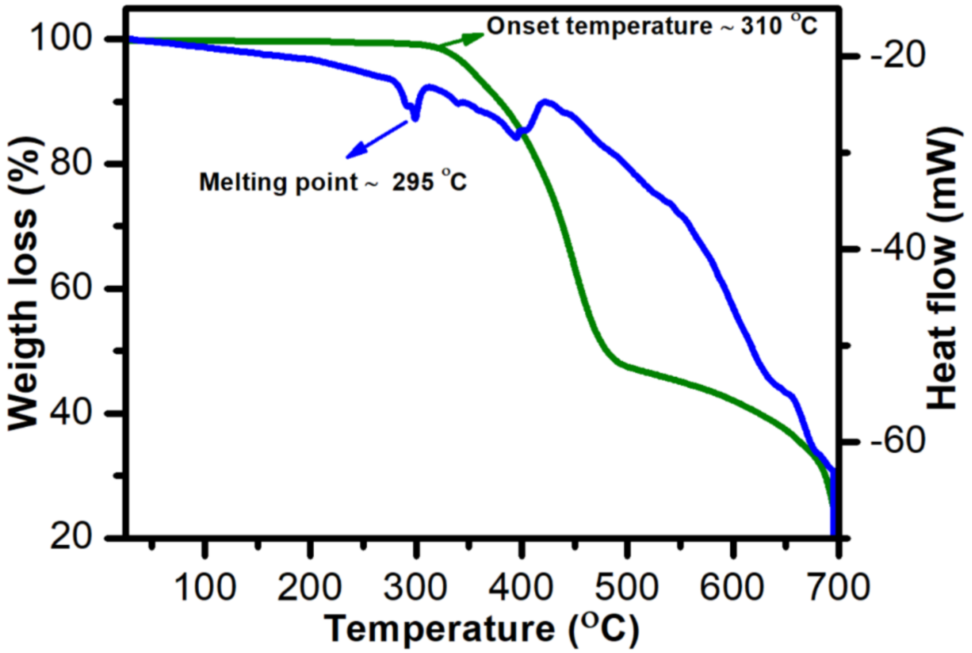

Supplementary Figure 3 | The thermogravimetric analysis (TGA) and differential scanning calorimetry (DSC) results of ($C_{38}H_{34}P_2$)$MnBr_4$.

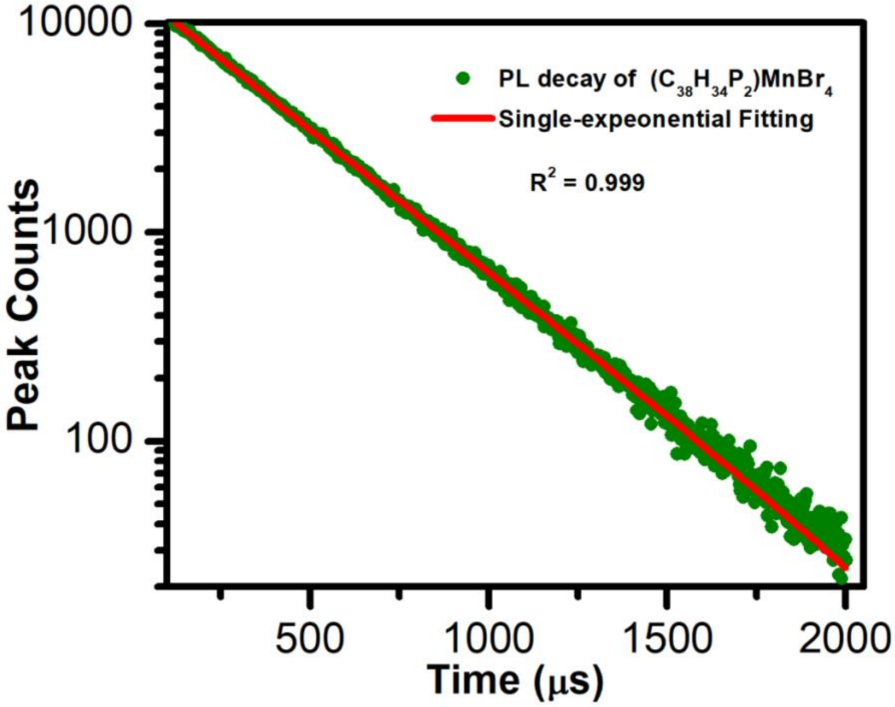

Supplementary Figure 4 | Time resolved photoluminescence of ($C_{38}H_{34}P_2$)$MnBr_4$ with a single exponential fitting (R_2 = 0.999).

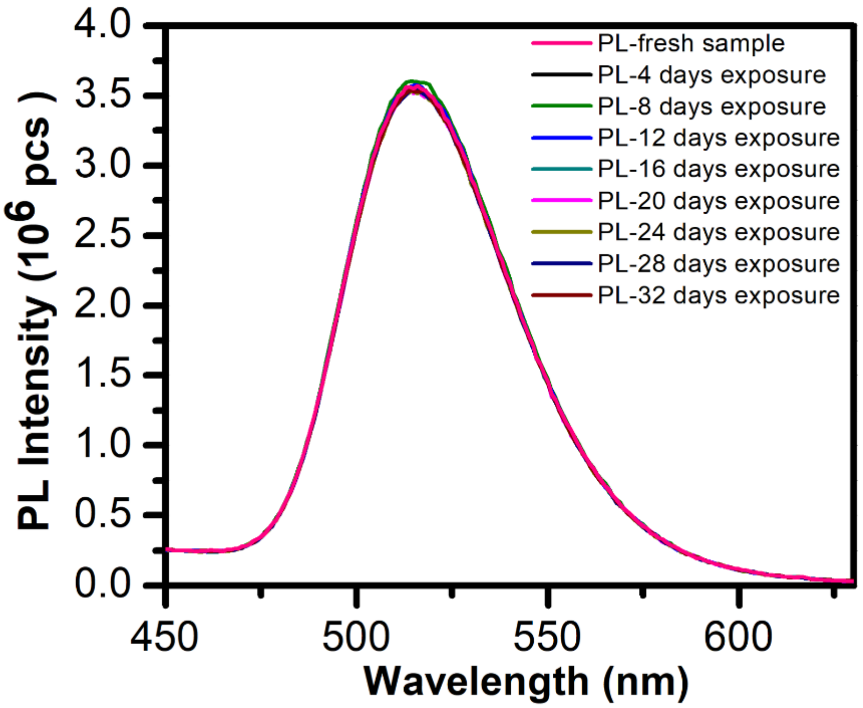

Supplementary Figure 5 | Photoluminescence intensities of ($C_{38}H_{34}P_2$)$MnBr_4$ at different exposure times in ambient atmosphere.

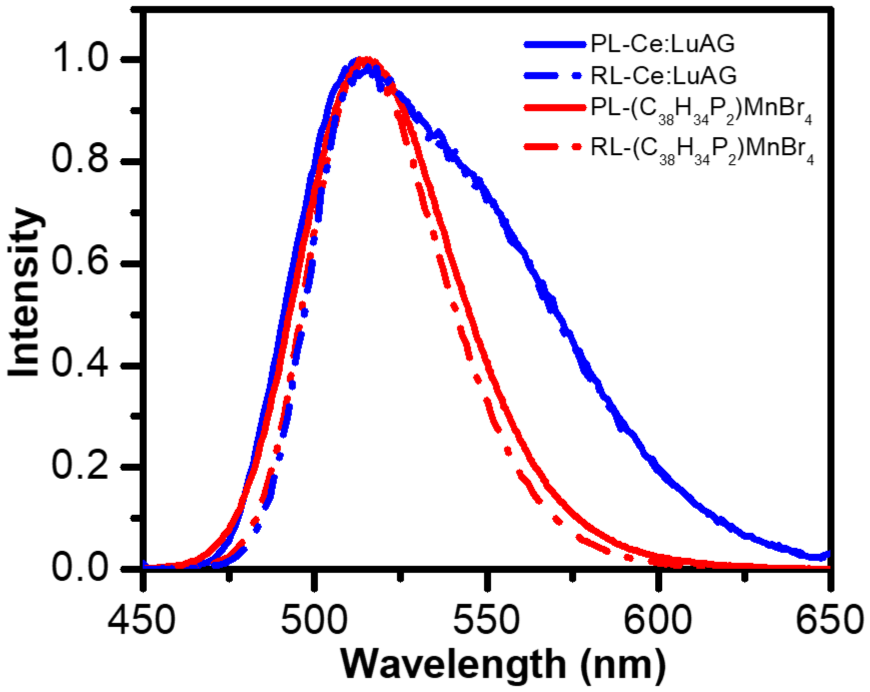

Supplementary Figure 6 | The radioluminescence and photoluminescence spectra of $(C_{38}H_{34}P_2)MnBr_4$ and Ce:LuAg.

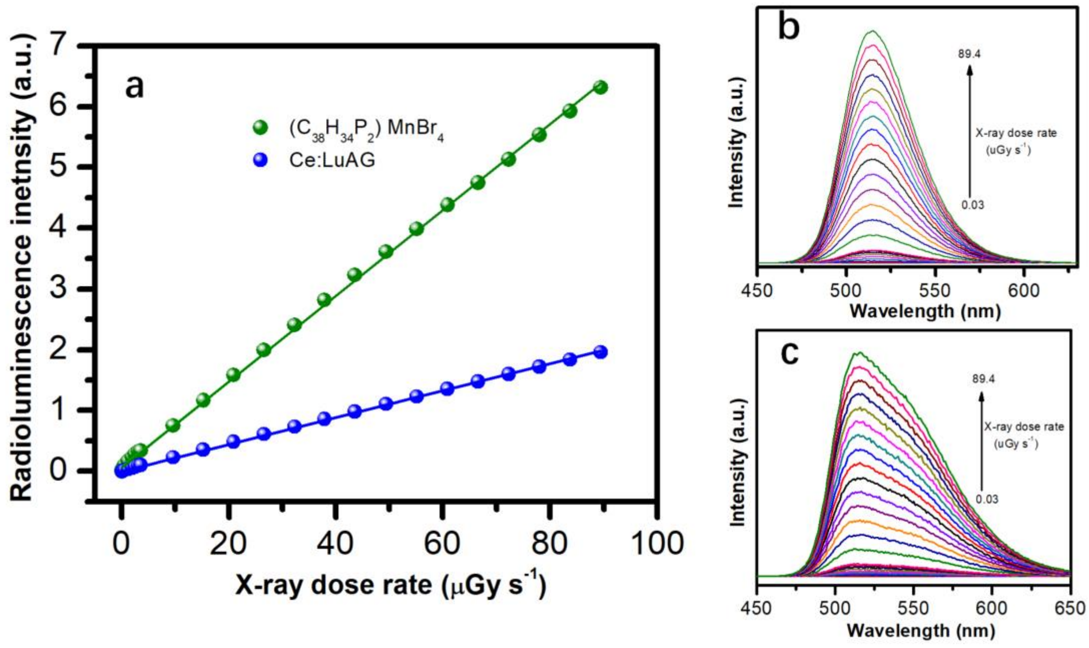

Supplementary Figure 7 | (a) Radioluminescence intensity of $(C_{38}H_{34}P_2)MnBr_4$ and Ce:LuAgLYSO as a linear response to X-ray dose rate in a wide range from 0.37 to 89.4 µGy s^{-1}. (b) X-ray dose rate dependent radioluminescence of $(C_{38}H_{34}P_2)MnBr_4$. (c). X-ray dose rate dependent radioluminescence of Ce:LuAG.

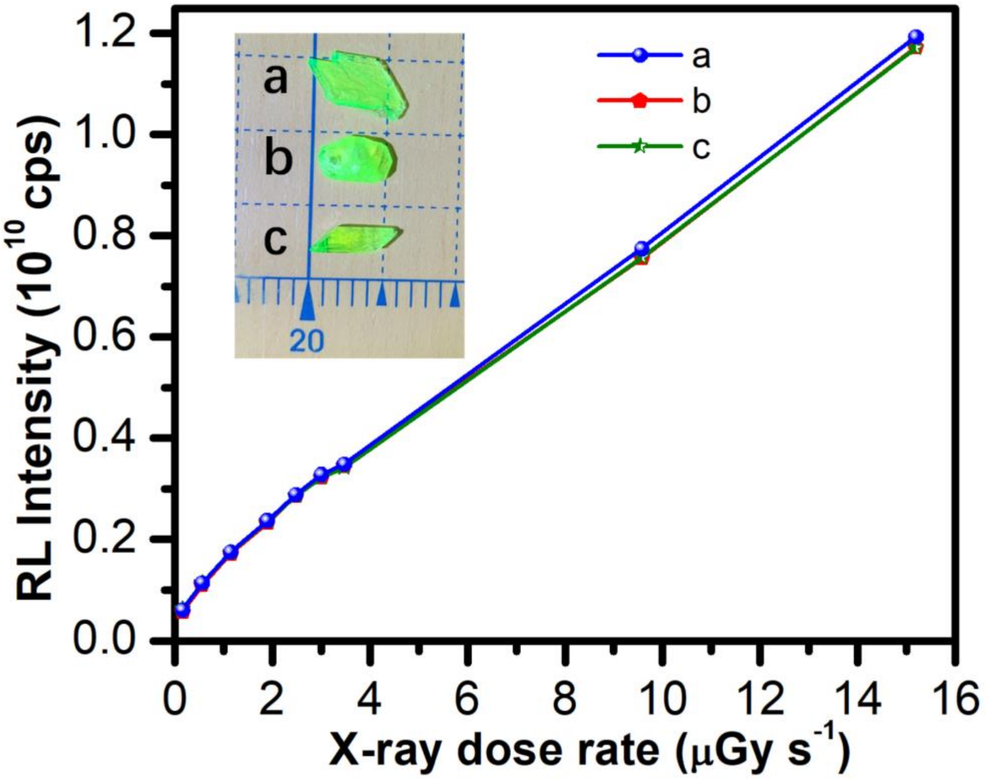

Supplementary Figure 8 | Dose rate dependence of the RL intensity of $(C_{38}H_{34}P_2)MnBr_4$ with different shapes and sizes. The results show these three cyrstals have almost the same response to X-ray dose rate.

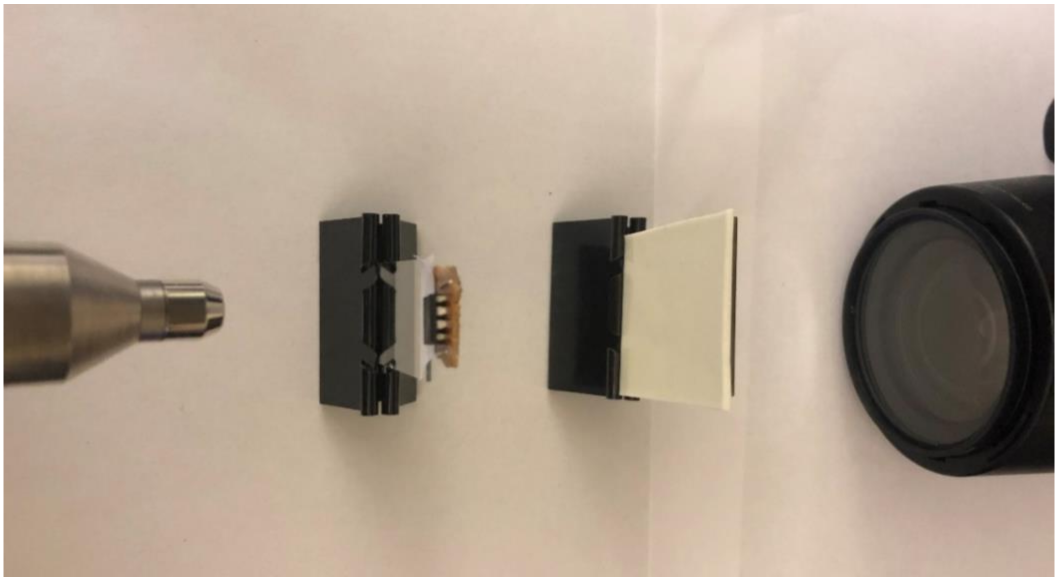

Supplementary Figure 9 | Projection configuration of a home-built X-ray imaging system.

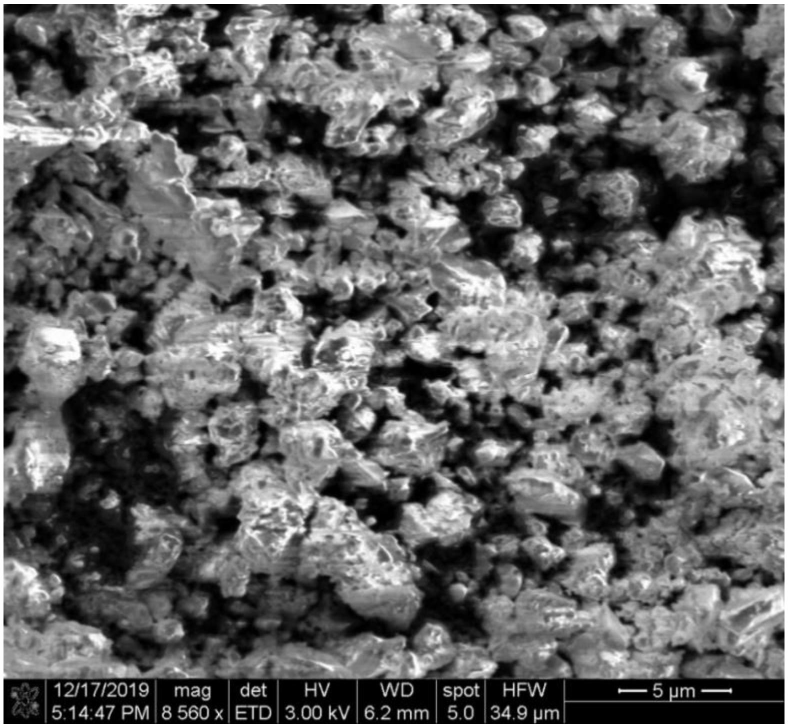

Supplementary Figure 10 | SEM image of ($C_{38}H_{34}P_2$)$MnBr_4$ fine powder.

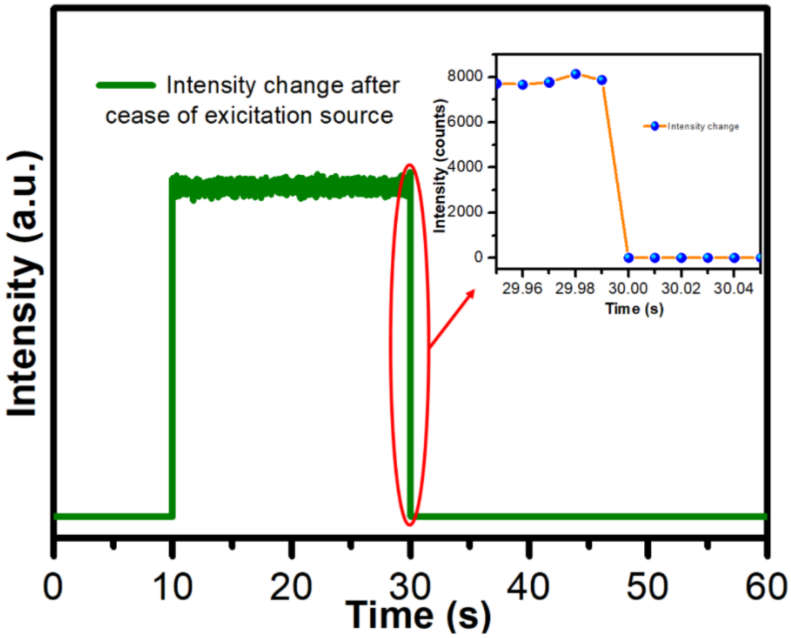

Supplementary Figure 11 | Afterglow intensities of ($C_{38}H_{34}P_2$)$MnBr_4$ after cease of excitation source at 30 s (the shutter is open at 10 s and closed at 30 s). The inset shows the details of intensity change with time interval of 10 ms which indicating no residual signal after 10 ms.

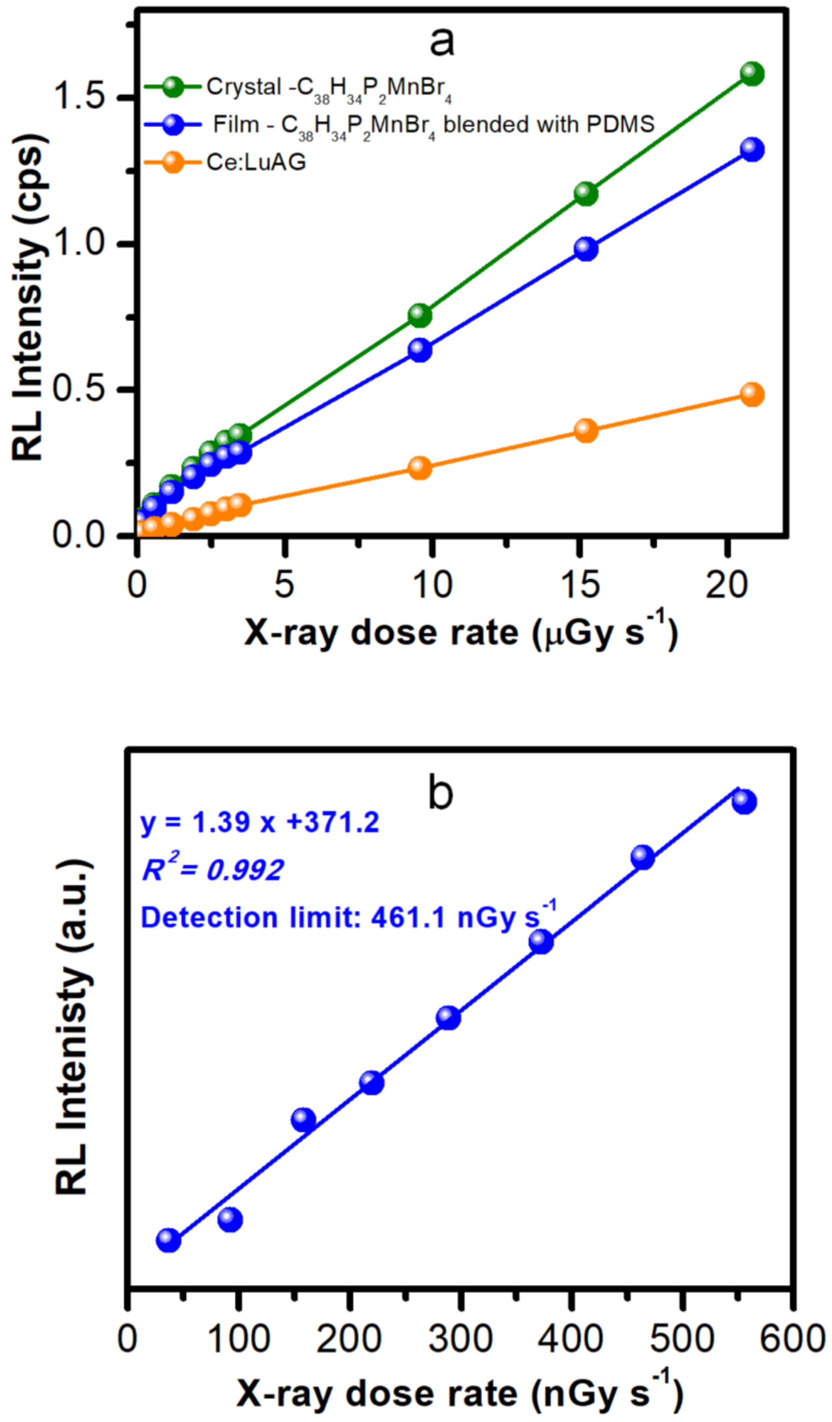

Supplementary Figure 12 | (a) Dose rate dependence of the RL intensity of standard reference Ce:LuAG, ($C_{38}H_{34}P_2$)$MnBr_4$ crystals, and ($C_{38}H_{34}P_2$)$MnBr_4$ in PDMS thin films. (b) The detection limit measurement under low X-ray dose for a flexible scintillator containing ($C_{38}H_{34}P_2$)$MnBr_4$.
