# Supplementary material for: Highly efficient eco-friendly X-ray scintillators based on an organic manganese halide
Source: Nat Commun. 2020 Aug 28;11:4329. doi: 10.1038/s41467-020-18119-y (PMC7455565; doi:10.1038/s41467-020-18119-y)
Supplement: Supplementary file 2 — Peer Review File [file 41467_2020_18119_MOESM2_ESM.docx]

Peer Review Information

**Journal:** Nature Communications

**Manuscript title:** Highly efficient eco-friendly X-ray scintillators based on an organic manganese halide

**Corresponding author name(s):** Prof Biwu Ma

**Editorial notes:**

**none**

**Reviewer comments & decisions:**

**Reviewer comments, first version:**

Reviewer #1 (Remarks to the Author):

The authors have demonstrated the synthesis and use of inch-sized zero-dimensional ethylenebis-triphenylphosphonium manganese (II) bromide ((C38H34P2)MnBr4) crystals as a strong scintillation material for potential application in x-ray detection and imaging technologies. This material demonstrates emission at 517 nm with a high PLQE of ~95%. The authors have shown the PLQE of this material to maintain stable even after exposure to moisture. Additionally, this material has demonstrated a linear response in radioluminescence within a dose range from 36.7 nGy/s to 89.4 nGy/s with a detection limit measured to be 72.8 nGy/s. (C38H34P2)MnBr4 also seems to have a great light yield of 79,800 Photon/MeV that is stable for over 2hrs at 26 μGy/s. These factors make this material particularly attractive for scintillation applications. The authors also demonstrate the attractive ability to mix powdered (C38H34P2)MnBr4 crystals with PDMS to make a flexible scintillator screen. The work, as presented here, seems attractive and has potential to be impactful; however, there are a number of questions that should be addressed and potential issue to be clarified, before I can recommend the publication of this manuscript in Nature Comm.


Comment 1.

In the abstract, the author claims to be able to achieve crystals on the order of inches; however, in the manuscript there is no evidence of crystals over even just 1 cm. Additionally, even if 1 inch is possible but the crystal is not of high enough quality to attain the same results as less than 1 cm, the claim of size is no longer an acceptable claim or important. This needs to be addressed.

Comment 2.

I feel the authors’ claim of the material being eco-friendly may not be fully justified. There is little information or study done on the effects on health or environment of ethylenebis-triphenylphosphonium bromide. The solvents used in crystallization, diethyl ether and DCM, are not entirely environmentally friendly, both with environmental toxicity similar to the levels of lead metal in some instances. This should be addressed or the wording of the claims must be changed.

Comment 3.

The author claims (C38H34P2)MnBr4 possesses high phases purity based on SCXRD and high thermally stability due to no weight loss in TGA up to 310 °C. However, the author does not show any information on the phase stability at elevated temperatures below 310 °C and if phase transitions alter the performance. Potentially determining the phase purity and performance within a reasonable window of operating temperatures as well as with thermal cycling could better support this claim of stability.


Comment 4.

The author tested the RL stability of (C38H34P2)MnBr4 under 26 μGy/s for 2 hours. Although the material appears stable in this time frame, it may be important to determine at what point there is a loss in performance.

Comment 5.

The authors demonstrate flexible scintillation screens made from ground crystals incorporated into PDMS, but offer none of the resulting properties that were reported for the single crystal. If there is a reduction in performance with the fabrication these screens, it should be reported.

Comment 6.

The authors make the claim of (C38H34P2)MnBr4 being capable of high-resolution X-ray imaging, but provides to quantitative value.

Comment 7.

Figure 2b, the author displays the RL intensity with respect to dose rate for (C38H34P2)MnBr4 and Ce:LuAG. The author claims for the response of (C38H34P2)MnBr4 to be linear, but in the main plot below 5 uGy/s the claim that the RL response of (C38H34P2)MnBr4 is linear here is questionable.

Comment 8.

The authors claim (C38H34P2)MnBr4 has a light yield of 79,800 Photon/MeV. They provide very limited information on the calculation of this value and seem to just approximate by multiplying the light yield of Ce:LuAG by the ratio of dose response of (C38H34P2)MnBr4 and Ce:LuAG ((C38H34P2)MnBr4/Ce:LuAG = 3.2). The author should clarify whether they accurately calculated this value by correcting for the wavelength dependent detection efficiency of the detector toward (C38H34P2)MnBr4 and Ce:LuAG.

Comment 9.

The authors present a detection limit of 72.8 nGy/s for (C38H34P2)MnBr4 scintillating crystals and claim this to be very good for Pb-free perovskite scintillating materials, which is true based on reported claims so far. The authors also use the light yield of (C38H34P2)MnBr4 (79,800 Photon/MeV) to distinguish it as a superior material; however, the full comparison to other Pb-free perovskite scintillating materials is not provided. In fact, Tang et al’s work (DOI: 10.1002/adma.201904711) demonstrates 1D Rb2CuBr3 to have a light yield of 91,056 Photons/MeV. It is important to be more comprehensive and clearer in comparisons with previous reports and commercial technologies.

Comment 10.

It needs to be clarified whether the results of this paper were obtained from just a single sample or whether there is good reproducibility between crystals grown the same way.


Comment 11.

There is a lack of details in the methods and supporting information regarding both the synthetic techniques as well as the testing and data processing. It is hard to determine whether the reported data is reliable and trustworthy without more detailed explanations of parameters used from synthesis to data processing.

Comment 12.

Minor grammatical and English language errors throughout manuscript.


Reviewer #2 (Remarks to the Author):

In their submitted manuscript, Xu et al. describe the use of a novel X-ray scintillating material, (C38H34P2)MnBr4. It can efficiently convert X-rays into visible light. A high light yield of ~ 80,000 photons/MeV, and a low detection limit of 72.8 nGy/s are reported in single crystals and said to be superior to those of today’s commercially available scintillators as well as other emerging scintillating materials. Dispersed as powder in a rubber material, a flexible scintillating sheet was made and used to record (high resolution) images.
The manuscript is well prepared and of appropriate length. It is well organized and clearly written. The work is of interest to a specialized audience in the field of X-ray scintillators; but not enough information is given to consider its competitiveness, most notably spatial resolution and time response.

Points 2-4 below require further clarification prior to this paper being considered for publication. The other points I see as less important.
1. The authors compare the scintillating properties of (C38H34P2)MnBr4 with cerium-doped lutetium aluminum garnet (Ce:LuAG). Why this material? To the best of my knowledge, scintillating X-ray imagers mostly use thallium-doped cesium iodide (CsI:Tl) or terbium doped gadolinium oxysulfide (Gd2O2S:Tb, GOS:Tb), placed on top of a photodiode detector array. I believe that the manuscript should benchmark (more) with respect to these materials.
2. Integration of single crystals is not low-cost. The use of powder-PDMS blend potentially is. However, this flexible scintillator screen is not properly characterized. Light yield, stopping power, dynamic range/linearity, … Are the properties of the powder and the crystal the same? See also next points
3. MTF measurements should be carried out to quantify the spatial resolution of the images. Here, the columnar structure of the CsI has a great advantage, the needle-like crystals result in a good (sharp) spatial resolution, as typically quantified in terms of MTF. Authors should show MTF (or similar) measurements for the images shown in the manuscript.
4. In order to obtain a reliable fast image readout, time-dependent light measurements are needed. Does the material (single crystal and powder) show a mono-exponential decay (measurement data in Figure S4 is not clear). Is there ghosting, (i.e. residual signal after 1s), resulting in possible image lag? Is the long decay time (compared to other scintillator materials) a problem for some applications?
5. Is the reported light yield (ph/MeV) independent of radiation energy? Typical energies used in (medical) X-ray detection is 20-100 keV. Authors are invited to elaborate if this is important.
6. Figure 2d. Why is the light output increasing with (prolonged) X-ray exposure?
7. The manganese material is claimed to be a eco-friendly alternative for existing scintillating materials. Authors are invited to clarify why their manganese-based scintillating materials are more eco-friendly than other (non-lead) organic metal halides such as those used in ref 31-33 and also the already mentioned CsI:Tl and Gd.
8. What was the solid state content of (C38H34P2)MnBr4 in the PDMS blend?


Reviewer #3 (Remarks to the Author):

The manuscript reports about a new 0D organic manganese (II) halide hybrid (C38H34P2)MnBr4 which exhibit highly efficient green emission upon photo and X-ray excitations. The manuscript is very well written and the experimental interpretation seems sound. I'm generally positive about the work and I will suggest its acceptance. I have, however, a suggestion aimed at making the work even more interesting. Would be interesting if the authors could give a reason for the high performance of this material, as this would help serching for more efficient scintillators.

**Author rebuttal, first version:**

Reviewer #1: The authors have demonstrated the synthesis and use of inch-sized zero-dimensional ethylenebis-triphenylphosphonium manganese (II) bromide ((C38H34P2)MnBr4) crystals as a strong scintillation material for potential application in x-ray detection and imaging technologies. This material demonstrates emission at 517 nm with a high PLQE of ~95%. The authors have shown the PLQE of this material to maintain stable even after exposure to moisture. Additionally, this material has demonstrated a linear response in radioluminescence within a dose range from 36.7 nGy/s to 89.4 nGy/s with a detection limit measured to be 72.8 nGy/s. (C38H34P2)MnBr4 also seems to have a great light yield of 79,800 Photon/MeV that is stable for over 2hrs at 26 μGy/s. These factors make this material particularly attractive for scintillation applications. The authors also demonstrate the attractive ability to mix powdered (C38H34P2)MnBr4 crystals with PDMS to make a flexible scintillator screen. The work, as presented here, seems attractive and has potential to be impactful; however, there are a number of questions that should be addressed and potential issue to be clarified, before I can recommend the publication of this manuscript in Nature Comm.

**Response: We thank the reviewer for the recommendation.**

Comment 1. In the abstract, the author claims to be able to achieve crystals on the order of inches; however, in the manuscript there is no evidence of crystals over even just 1 cm. Additionally, even if 1 inch is possible but the crystal is not of high enough quality to attain the same results as less than 1 cm, the claim of size is no longer an acceptable claim or important. This needs to be addressed.

**Response: Actually, we have obtained crystals with 1 inch long as shown in following figure and replaced the previous figure 1c,1d with them in the revised manuscript. Since the crystals have near unity PLQEs, and all the crystals with sufficient thickness to absorb X-ray effectively, they would exhibit the same scintillation performance. In the revision, we performed the reproducibility of the response to X-ray on (C38H34P2)MnBr4 by using crystals with different sizes and shapes. As shown in Figure S8 in the revised supporting information, all the crystals exhibit almost the same sensitivity to X-ray.**

**Figure** (a) Inch sized (C38H34P2)MnBr4 crystal under bright field. (b) Inch sized (C38H34P2)MnBr4 crystal under UV light.

**Figure S8.** Dose rate dependence of the RL intensity of (C38H34P2)MnBr4 with different shape and size. The results show these three crystals have almost the same response to X-ray dose rate.

Comment 2. I feel the authors’ claim of the material being eco-friendly may not be fully justified. There is little information or study done on the effects on health or environment of ethylenebis-triphenylphosphonium bromide. The solvents used in crystallization, diethyl ether and DCM, are not entirely environmentally friendly, both with environmental toxicity similar to the levels of lead metal in some instances. This should be addressed or the wording of the claims must be changed.

**Response: We agrees with the reviewer that the solvents (diethyl ether and DCM) used in crystallization are not environmentally friendly. Indeed, the synthesis procedure could be modified by using other environment-friendly solvents. For instance, we can use dimethylformamide (DMF) to obtain high quality crystals by cooling down the preheated DMF solution containing the precursors. Here, the claim of the material being eco-friendly is mainly focusing on the final product itself, as compared to other metal containing scintillators. In the revision, we have provided more details regarding the toxicity of metal halides and other materials. The toxicity classification (health and environment) of the metal halides was acquired from material safety data sheet (MSDS). As shown in the following table, Mn(II) is relatively friendly among the reported and**

**commercially available scintillators. In the revision, we added a new paragraph. “Moreover, based on the toxicity classification (health and environment) information of metal halides from material safety data sheet (MSDS), (C38H34P2)MnBr4 is believed to be significantly less toxic than existing scintillators mentioned above. As shown in Table S3, Pb(II), Cu(I), CsI and GdWO4 possess the most severe toxicity in environment, and Tl(I) and CsI are moderately toxic to health. Also 87Rb isotope is radioactive. Mn(II) is considered to be low toxic for health and friendly in environment.” In the revised supporting information, we added the table S3 as following.**

**Table. S3** The toxicity classification (health and environment**)** of the metal halides acquired from material safety data sheet (MSDS). Acute toxicity category 1 represents the most severe toxicity with LD50 < 5 mg/kg in oral, and category 2 with 5< LD50 < 50 mg/kg, category 3 with 50 < LD50 < 300 mg/kg and category 4 with 300 < LD50 < 2000 mg/kg. As the toxicity classification of the reported and commercially available scintillator are unknow, we compare the toxicity of metal halides used for scintillators synthesis for convenience**.** Also, we refer to the toxicity of tetraphenylphosphonium bromide instead of ethylenebis-triphenylphosphonium bromide as its MSDS data is unavailable.

| **Materials** | **Acute toxicity (Health)** | **Acute aquatic hazard**  **(Environment)** |
| --- | --- | --- |
| PbBr2 | Category 4 | Category 1 |
| CuCl | Category 4 | Category 1 |
| TlBr | Category 2 | Category 2 |
| CsI | Category 2 | Category 1 |
| CdWO4 | Category 4 | Category 1 |
| MnBr2 | Category 4 | Contains no substances known  to be hazardous to the  environment |
| TPPBr  (Tetraphenylphosphonium  bromide) | Not a hazardous substance | Not a hazardous substance |

Comment 3.The author claims (C38H34P2)MnBr4 possesses high phases purity based on SCXRD and high thermally stability due to no weight loss in TGA up to 310 °C. However, the author does not show any information on the phase stability at elevated temperatures below 310 °C and if phase transitions alter the performance. Potentially determining the phase purity and performance within a reasonable window of operating temperatures as well as with thermal cycling could better support this claim of stability.

**Response: Following the reviewer’s suggestion, we performed the TG-DSC measurement on (C38H34P2)MnBr4 , and an endothermic peak at 295 °C was observed, which could be the melting point of (C38H34P2)MnBr4. The results suggest that there are unlikely phase transitions below 295 °C. In our revised Supporting Information, we added the following figure as Figure S3. In the revised manuscript, we added a new sentence: “The differential scanning calorimetry (DSC) results with an endothermic peak at 295 °C (Figure S3), which could be the melting point of (C38H34P2)MnBr4, suggest its high phase stability at elevated temperatures below 295 °C.”**

**Figure S3.** The thermogravimetric analysis (TGA) and differential scanning calorimetry (DSC) results of (C38H34P2)MnBr4.

Comment 4.The author tested the RL stability of (C38H34P2)MnBr4 under 26 μGy/s for 2 hours. Although the material appears stable in this time frame, it may be important to determine at what point there is a loss in performance.

**Response: As suggested by the reviewer, we repeated the stability measurement under the same X-ray dose rate (26 μGy/s) for a longer time of 4h, no change of the intensity was observed. Then we increased the X-ray dose rate to 89.4 μGy/s, little to no RL intensity change was observed after exposure for 4h as well. These results are not surprising as (C38H34P2)MnBr4 is highly emissive with near unity PLQE and stable with high decomposition temperature. In the revised manuscript, we updated the Figure 2d as shown below and revised the main text: “The stability of (C38H34P2)MnBr4 single**

**crystals against X-ray irradiation was evaluated by monitoring the changes of RL intensity under continuous X-ray irradiation with a dose rate of 89.4 μGy/s. Figure 2d shows that little-to-no radio-degradation was observed after 4h exposure to X-ray irradiation, suggesting high stability for scintillator applications.”**

**Figure 2d.** The change of the RL intensity under continuous X-ray excitation with a dose rate of 89.4 μGy/s.

Comment 5.The authors demonstrate flexible scintillation screens made from ground crystals incorporated into PDMS, but offer none of the resulting properties that were reported for the single crystal. If there is a reduction in performance with the fabrication these screens, it should be reported.

**Response: Similar to single crystals, we have characterized the linearity to X-ray dose rate, light yield, and detection limit on the flexible scintillation screens. The results (Figure S12) show the overall performance of the blend is just a little bit inferior to that of single crystals. This is not surprising, considering that the content of (C38H34P2)MnBr4 is reduced in the blends, the distribution of (C38H34P2)MnBr4 might not be perfectly uniform in the blends, and PDMS could also affect the X-ray absorption. In the revised manuscript, we added the performance of flexible scintillation screens. “The scintillation performance of flexible scintillation screens was characterized as**

**shown in Figure S12, which exhibit excellent linearities to the X-ray dose rates in a large range from 36.7nGy/s to 89.4 μGy/s, with a slightly lower light yield (66,256 photon/MeV) and detection limit (461.1 nGy/s), as compared to those of single crystals. This is not surprising, considering that the content of (C38H34P2)MnBr4 is reduced in the blends, the distribution of (C38H34P2)MnBr4 might not be perfectly uniform in the blends, and PDMS could also affect the X-ray absorption.” In the revised Supporting Information, we provided the performance the flexible scintillation screens as shown in Figure S12.**

**Figure S12.** (a) Dose rate dependence of the RL intensity of standard reference Ce:LuAG, (C38H34P2)MnBr4 crystals, and (C38H34P2)MnBr4 in PDMS thin films. (b) The detection limit measurement under low X-ray dose for a flexible scintillator containing (C38H34P2)MnBr4.

Comment 6.The authors make the claim of (C38H34P2)MnBr4 being capable of high-resolution X-ray imaging, but provides to quantitative value.

**Response: As suggested by the reviewer, we measured the X-ray imaging resolution of (C38H34P2)MnBr4, by fitting the point spread function of the intensity profile with a Gaussian function and obtained a spatial resolution as high as 0.322 mm. The corresponding description and Figure were added and highlighted in the revised manuscripts as “The spatial resolution was calculated as 0.322 mm by fitting the point spread function of the intensity profile (Figure 2g).”**

**Figure 2g.** Spatial resolution measurement by fitting of intensity spread profile with Gaussian function. The FWHM was taken as resolution. The red line in Figure 2f shows the data trace of collection.

Comment 7. Figure 2b, the author displays the RL intensity with respect to dose rate for (C38H34P2)MnBr4 and Ce:LuAG. The author claims for the response of (C38H34P2)MnBr4 to be linear, but in the main plot below 5 uGy/s the claim that the RL response of (C38H34P2)MnBr4 is linear here is questionable.

**Response: We evaluated the linearity of the RL response of (C38H34P2)MnBr4 below 5 μGy/s as shown below, which showed a good linearity to the X-ray dose rate with R2 as 0.985.**

0.0 0.5 1.0 1.5 2.0 2.5 3.0 3.5 **X-ray dose rate (μGy/s)**

**Radioluminescence Intensity (a.u.)**

**Linear Fitting**

___

**^(C^38^H^34^)P^2^MnBr^4**

R2 = 0.985

Comment 8.The authors claim (C38H34P2)MnBr4 has a light yield of 79,800 Photon/MeV. They provide very limited information on the calculation of this value and seem to just approximate by multiplying the light yield of Ce:LuAG by the ratio of dose response of (C38H34P2)MnBr4 and Ce:LuAG ((C38H34P2)MnBr4/Ce:LuAG = 3.2). The author should clarify whether they accurately calculated this value by correcting for the wavelength dependent detection efficiency of the detector toward (C38H34P2)MnBr4 and Ce:LuAG.

**Response: The details of the light yield calculation has been provided in the method section in the revised manuscript. Here, the X-ray response intensity was examed and collected by a Hamamatsu R928 PMT and the spectra were corrected for intensity and wavelength from Edinburgh Instruments provided correction files automatically. Since the spectra of (C38H34P2)MnBr4 is similar to that of Ce:LuAG after correcting by R928 PMT, the light yield could be estimated by comparing the corrected response amplitude (R) of the two samples based on the equation as follows.**

Comment 9.The authors present a detection limit of 72.8 nGy/s for (C38H34P2)MnBr4 scintillating crystals and claim this to be very good for Pb-free perovskite scintillating materials, which is true based on reported claims so far. The authors also use the light yield of (C38H34P2)MnBr4 (79,800 Photon/MeV) to distinguish it as a superior material; however, the full comparison to other Pb-free perovskite scintillating materials is not provided. In fact, Tang et al’s work (DOI: 10.1002/adma.201904711) demonstrates 1D Rb2CuBr3 to have a light yield of 91,056 Photons/MeV. It is important to be more comprehensive and clearer in comparisons with previous reports and commercial technologies.

**Response: As suggested by the reviewer, we made a comparison of light yield between our material and previously reported ones, including Rb2CuBr3, Rb2CuCl3 and Cs3Cu2I5 NCs and commercially available scintillators, as shown in the following figure. In the revised manuscript, we added a new paragraph. “As shown in Figure 2c, the light yield of (C38H34P2)MnBr4 is comparable to those of recently reported lead-free metal halides, such as Cs3Cu2I5 (79279 photon/MeV) and Rb2CuBr3 (91056 photon/MeV), and much better than those of Rb2CuCl3 (16600 photon/MeV), widely investigated CsPbBr3 nanocrystals (21000 photon/MeV), and many commercially available scintillators, such as CsI(Tl) and CdWO4.” We have updated the figure 2c as follows.**

**Figure 2c.** Comparison of scintillator light yields of (C38H34P2)MnBr4 and previously reported and commercially available scintillators.

Comment 10.It needs to be clarified whether the results of this paper were obtained from just a single sample or whether there is good reproducibility between crystals grown the same way.

**Response: The reproducibility was verified by measuring the X-ray dose rate dependent RL intensity on crystals with different sizes and shapes. The results as shown below exhibit great reproducibility for different crystals. In the revised manuscript, we added “The reproducibility of the responses to X-ray for (C38H34P2)MnBr4 was validated by using single crystals with different sizes and shapes. Almost the same sensitivity was recorded for all the samples, as shown in Figure S8.” In the revised supporting information, we added the following figure as Figure S8.**

**Figure S8.** Dose rate dependence of the RL intensity of (C38H34P2)MnBr4 with different shape and size. The results show these three crystals have almost the same response to X-ray dose rate.

Comment 11. There is a lack of details in the methods and supporting information regarding both the synthetic techniques as well as the testing and data processing. It is hard to determine whether the reported data is reliable and trustworthy without more detailed explanations of parameters used from synthesis to data processing.

**Response: More details of the synthesis and data processing were provided and highlighted in method section in the revised manuscripts.**

Comment 12. Minor grammatical and English language errors throughout manuscript.

**Response: We have revised the manuscript accordingly to avoid typos and grammar errors.**

Reviewer #2:

In their submitted manuscript, Xu et al. describe the use of a novel X-ray scintillating material, (C38H34P2)MnBr4. It can efficiently convert X-rays into visible light. A high light yield of ~ 80,000 photons/MeV, and a low detection limit of 72.8 nGy/s are reported in single crystals and said to be superior to those of today’s commercially available scintillators as well as other emerging scintillating materials. Dispersed as powder in a rubber material, a flexible scintillating sheet was made and used to record (high resolution) images.

The manuscript is well prepared and of appropriate length. It is well organized and clearly written. The work is of interest to a specialized audience in the field of X-ray scintillators; but not enough information is given to consider its competitiveness, most notably spatial resolution and time response.

Points 2-4 below require further clarification prior to this paper being considered for publication. The other points I see as less important.

**Response: We thank the reviewer for the positive comments and suggestions.**

1. The authors compare the scintillating properties of (C38H34P2)MnBr4 with cerium-doped lutetium aluminum garnet (Ce:LuAG). Why this material? To the best of my knowledge, scintillating X-ray imagers mostly use thallium-doped cesium iodide (CsI:Tl) or terbium doped gadolinium oxysulfide (Gd2O2S:Tb, GOS:Tb), placed on top of a photodiode detector array. I believe that the manuscript should benchmark (more) with respect to these materials.

**Response: We chose Ce:LuAG as the reference, as it has similar photoluminescence and radioluminescence as those of (C38H34P2)MnBr4, which would minimize the influence of response difference by detectors. Also, we indeed compared the light yields of various scintillators, including CsI:Tl, LYSO and so on. (Figure 2c)**

2. Integration of single crystals is not low-cost. The use of powder-PDMS blend potentially is. However, this flexible scintillator screen is not properly characterized. Light yield, stopping power, dynamic range/linearity, ... Are the properties of the powder and the crystal the same? See also next points

**Response: Similar to single crystals, we have characterized the linearity to X-ray dose rate, light yield, and detection limit on the flexible scintillation screens. The results (Figure S12) show the overall performance of the blend is just a little bit inferior to that of single crystals. This is not surprising, considering that the content of (C38H34P2)MnBr4 is reduced in the blends, the distribution of (C38H34P2)MnBr4 might not be perfectly uniform in the blends, and PDMS could also affect the X-ray absorption. In the revised manuscript, we added the performance of flexible scintillation screens.**

**“The scintillation performance of flexible scintillation screens was characterized as shown in Figure S12, which exhibit excellent linearities to the X-ray dose rates in a large range from 36.7nGy/s to 89.4 μGy/s with a slightly lower light yield (66256 photon/MeV) and detection limit (461.1 nGy/s), as compared to those of single crystals. This is not surprising, considering that the content of (C38H34P2)MnBr4 is reduced in the blends, the distribution of (C38H34P2)MnBr4 might not be perfectly uniform in the blends, and PDMS could also affect the X-ray absorption.” In the revised Supporting Information, we provided the performance the flexible scintillation screens as shown in Figure S12.**

**Figure S12.** (a) Dose rate dependence of the RL intensity of standard reference Ce:LuAG and (C38H34P2)MnBr4 crystal and (C38H34P2)MnBr4 film blended with PDMS. (b) The detection limit measurement under low X-ray dose for (C38H34P2)MnBr4 film blended with PDMS.

3. MTF measurements should be carried out to quantify the spatial resolution of the images. Here, the columnar structure of the CsI has a great advantage, the needle-like crystals result in a good (sharp) spatial resolution, as typically quantified in terms of MTF. Authors should show MTF (or similar) measurements for the images shown in the manuscript.

**Response: As suggested by the reviewer, we measured the X-ray imaging resolution of (C38H34P2)MnBr4, by fitting the point spread function of the intensity profile with a Gaussian function and obtained a spatial resolution as high as 0.322 mm. The corresponding description and Figure were added and highlighted in the revised manuscripts as “the spatial resolution was calculated as 0.322 mm by fitting the point spread function of the intensity profile (Figure 2g).”**

**Figure 2g.** Spatial resolution measurement by fitting of intensity spread profile with Gaussian function. The FWHM was taken as resolution. The red line in Figure 2f shows the data trace of collection.

4. In order to obtain a reliable fast image readout, time-dependent light measurements are needed. Does the material (single crystal and powder) show a mono-exponential decay (measurement data in Figure S4 is not clear). Is there ghosting, (i.e. residual signal after 1s), resulting in possible image lag? Is the long decay time (compared to other scintillator materials) a problem for some applications?

**Response: Following the reviewer’s suggestion, we measured the afterglow intensity of the crystals as shown below. The intensity reduced to the background level at 10 ms after cease of the excitation source, which indicates no existence of ghosting and image lag based on this scintillator and it would not be a problem for practical applications. The updated lifetime result (Figure S4) show a mono-exponential decay for the crystals of (C38H34P2)MnBr4. In the revised manuscript, we added the related content and highlighted the sentence, “a high PLQE of ~ 95 % and a long single exponential decay lifetime of 318 µs (R2 = 0.999) (Figure S4).” “Image contrast is another important parameter for practical imaging applications, image lag or ghosting would happen if the emission with a long lifetime has a strong afterglow after X-ray being turned off. To exclude the effect of afterglow, we measured the afterglow**

**intensities of (C38H34P2)MnBr4, as shown in Figure S11. The intensity decreased to the background level in 10 ms after cease of the excitation source, indicating the suitability for high contrast imaging.” In the revised supporting information, we added the Figure S4 and Figure S11 as following.**

**Figure S4.** Time resolved photoluminescence of (C38H34P2)MnBr4 with single exponential fitting (R2 = 0.999)

**Figure S11.** Afterglow intensities of (C38H34P2)MnBr4 crystals after cease of excitation source at 30 s (the shutter is open at 10 s and then closed at 30 s). The inset shows the details of intensity change with time interval of 10 ms which indicating no residual signal after 10 ms.

5. Is the reported light yield (ph/MeV) independent of radiation energy? Typical energies used in (medical) X-ray detection is 20-100 keV. Authors are invited to elaborate if this is important.

**Response: The light yield (LY) of a scintillator represents a fraction of the visible photons generated after high-energy photon absorption. Herein, the number of UV–vis photons (Nph) produced in the scintillation conversion per energy E of incoming X(γ)-ray photon can be expressed as:**

**where *Eg* represents the forbidden gap of the material, *S* and *Q* are the quantum efficiencies of the transport and luminescence stages, respectively, and β is a phenomenological parameter which is typically found to be between 2 and 3 for most materials. (*Adv. Optical Mater.* 2015, *3*, 463–481) From the equation, it is reasonable to state that the light yield is only determined by the properties of the scintillation materials. Therefore, the light yield is independent of radiation energy.**

6. Figure 2d. Why is the light output increasing with (prolonged) X-ray exposure?

**Response: The slight increasing of the radioluminescence was likely due to the instrumental errors. We repeated the stability measurement by increasing the dose rate of X-ray light source to 89.4 μGy/s. Little to no RL intensity change was observed during the exposure, which is not surprising, as the (C38H34P2)MnBr4 is highly emissive with near unity PLQE and stable with high decomposition temperature. In the revised manuscript, we updated the Figure 2d as shown below and modified the related content and marked in this sentence: “The stability of (C38H34P2)MnBr4 single crystals against X-ray irradiation was evaluated by monitoring the changes of RL intensity under continuous X-ray irradiation with a dose rate of 89.4 μGy/s. Figure 2d shows that little to no radio-degradation was observed after 4h exposure to X-ray irradiation, suggesting high stability for scintillator application.”**

**Figure 2d.** The change of the RL intensity under continuous X-ray excitation with dose rate of 89.4 μGy/s.

7. The manganese material is claimed to be an eco-friendly alternative for existing scintillating materials. Authors are invited to clarify why their manganese-based scintillating materials are more eco-friendly than other (non-lead) organic metal halides such as those used in ref 31­33 and also the already mentioned CsI:Tl and Gd.

**Response: Here, the claim of the material being eco-friendly is mainly focusing on the final product itself, as compared to other metal containing scintillators. In the revision, we have provided more details regarding the toxicity of metal halides and other materials. The toxicity classification (health and environment) of the metal halides was acquired from material safety data sheet (MSDS). As shown in the following table, Mn(II) is relatively friendly among the reported and commercially available scintillators. In the revision, we added a new paragraph. “Moreover, based on the toxicity classification (health and environment) information of metal halides from material safety data sheet (MSDS), (C38H34P2)MnBr4 is believed to be significantly less toxic than existing scintillators mentioned above. As shown in Table S3, Pb(II), Cu(I), CsI and GdWO4 possess the most severe toxicity in environment, and Tl(I) and CsI are moderately toxic to health. Also 87Rb isotope is radioactive. Mn(II) is considered to be low toxic for health and friendly in environment.” In the revised supporting information, we added the table S3 as following.**

**Table. S3** The toxicity classification (health and environment**)** of the metal halides acquired from material safety data sheet (MSDS). Acute toxicity category 1 represents the most severe toxicity with LD50 < 5 mg/kg in oral, and category 2 with 5< LD50 < 50 mg/kg, category 3 with 50 < LD50 < 300 mg/kg and category 4 with 300 < LD50 < 2000 mg/kg. As the toxicity classification of the reported and commercially available scintillator are unknow, we compare the toxicity of metal halides used for scintillators synthesis for convenience**.** Also, we refer to the toxicity of tetraphenylphosphonium bromide instead of ethylenebis-triphenylphosphonium bromide as its MSDS data is unavailable.

| **Materials** | **Acute toxicity (Health)** | **Acute aquatic hazard**  **(Environment)** |
| --- | --- | --- |
| PbBr2 | Category 4 | Category 1 |
| CuCl | Category 4 | Category 1 |
| TlBr | Category 2 | Category 2 |
| CsI | Category 2 | Category 1 |
| CdWO4 | Category 4 | Category 1 |
| MnBr2 | Category 4 | Contains no substances known  to be hazardous to the  environment |
| TPPBr  (Tetraphenylphosphonium  bromide) | Not a hazardous substance | Not a hazardous substance |

8. What was the solid state content of (C38H34P2)MnBr4 in the PDMS blend?

**Response: The optimized content of (C38H34P2)MnBr4 is around 40 wt% and stated in the method section in the revised manuscript.**

Reviewer #3:

The manuscript reports about a new 0D organic manganese (II) halide hybrid (C38H34P2)MnBr4 which exhibit highly efficient green emission upon photo and X-ray excitations. The manuscript is very well written and the experimental interpretation seems sound. I'm generally positive about the work and I will suggest its acceptance. I have, however, a suggestion aimed at making the work even more interesting. Would be interesting if the authors could give a reason for the high performance of this material, as this would help serching for more efficient scintillators.

**Response: Together with previous reports (*Advanced Materials* 2019, 31,1904711; *The journal of physical chemistry letters* 2020, 11,1873-1880; *Adv Sci* 2020, 7, 2000195 and *ACS Materials Letters* 2020, 2, 633-638.) and our results, we believe that the negligible reabsorption and high PLQE are responsible for the high scintillation performance. And we mentioned it in the revised manuscript as “The excellent performance of X-ray imaging could be attributed to the negligible self-absorption, high PLQE, light yield, and low detection limit of (C38H34P2)MnBr4.”**

**Reviewer comments, second version:**

REVIEWERS' COMMENTS:

Reviewer #1 (Remarks to the Author):

The reviewer address my comments properly and I donot have anymore concern.


Reviewer #2 (Remarks to the Author):

The authors fully addressed my comments and improved the manuscript further. I suggest to publish the manuscript.

**Author rebuttal, second version:**

**Reviewer comments, third version:**

**Author rebuttal, third version:**
